# A nomogram to predict the risk of insulin resistance in Chinese women with polycystic ovary syndrome

**DOI:** 10.3389/fendo.2024.1446827

**Published:** 2024-11-27

**Authors:** Benjie Guo, Yuting Shen, Ziying Dai, Kalibinuer Yimamu, Jianhua Sun, Lixia Pei

**Affiliations:** Jiangsu Provincial Hospital of Traditional Chinese Medicine, Affiliated Hospital of Nanjing University of Traditional Chinese Medicine, Nanjing, China

**Keywords:** polycystic ovary syndrome, insulin resistance, PCOS combined with IR, nomogram, risk stratification

## Abstract

**Background:**

Insulin resistance (IR) is considered a major driver of the pathophysiology of polycystic ovary syndrome (PCOS), mediating the progression of hyperandrogenism and metabolic and reproductive dysfunction in patients with PCOS. Early detection of the risk of concurrent IR is essential for women with PCOS. To address this need, this study developed a predictive nomogram for assessing the risk of IR in women with PCOS, aiming to provide a tool for risk stratification and assist in clinical decision-making.

**Methods:**

Patients with untreated PCOS-IR diagnosed in a single-center retrospective cohort study from January 2023 to December 2023 were included for nomogram construction and validation. The area under the ROC curve (AUC), calibration curve, Hosmer–Lemeshow (H-L) goodness-of-fit test, and decision curve analysis (DCA) were used to evaluate the nomogram’s discrimination, calibration, and clinical decision performance. A risk stratification model based on the nomogram was then developed.

**Results:**

A total of 571 patients were included in the study; 400 patients enrolled before September 2023 were divided into the training and validation sets, and 171 patients enrolled later were used as the external validation set. The variables identified by logistic regression and the random forest algorithm—body mass index (BMI, OR 1.43), triglycerides (TG, OR 1.22), alanine aminotransferase (ALT, OR 1.03), and fasting plasma glucose (FPG, OR 5.19)—were used to build the nomogram. In the training, internal validation, and external validation sets, the AUCs were 0.911 (95% CI 0.878–0.911), 0.842 (95% CI 0.771–0.842), and 0.901 (95% CI 0.856–0.901), respectively. The nomogram showed good agreement between predicted and observed outcomes, and patients were categorized into low-, medium-, and high-risk groups based on their scores.

**Conclusions:**

Independent predictors of untreated PCOS-IR risk were incorporated into a nomogram that effectively classifies patients into risk groups, providing a practical tool for guiding clinical management and early intervention.

## 1 Introduction

Polycystic ovary syndrome (PCOS) is the most common endocrine and metabolic disorder in women of reproductive age, with a prevalence of 5%–18%, affecting women throughout their life cycle (1). Insulin resistance (IR) refers to the reduced ability of insulin to mediate metabolic effects like glucose uptake and lipolysis, requiring higher insulin levels (2). Between 44% and 70% of women with PCOS experience significant IR (3), with obese individuals at even higher risk (4). IR drives PCOS pathophysiology, contributing to hyperandrogenism, metabolic dysfunction, and reproductive disorders through various mechanisms (5). Women with PCOS and IR (PCOS-IR) face greater risk of adverse pregnancy outcomes and chronic conditions like cardiovascular disease and type 2 diabetes, creating substantial health and economic burdens (6). Early identification of IR risk is therefore crucial to improving clinical outcomes.

The hyperinsulinemic–euglycemic clamp (HIEC) is the gold standard for assessing IR but is costly and complex, limiting its use in clinical practice, especially in settings with limited resources (7). Simpler alternatives, such as the HOMA-IR and QUICKI indices (8), require fasting insulin measurements (9), which are often unavailable in primary care, particularly in underserved regions.

Several predictive models for PCOS-IR exist, incorporating biomarkers and clinical measures. Fulghesu et al. (10) explored urinary metabolite profiles correlated with hyperinsulinemia, suggesting a non-invasive approach, but this method requires specialized laboratory equipment, limiting its use in primary care. Li et al. (11) identified DIAPH1 as a biomarker for IR in PCOS, but this test is not widely accessible. Cree-Green et al. (12) developed a model based on clinical measures such as BMI and waist circumference, combined with the e-IS index, but it still depends on fasting insulin levels.

Primary care organizations, particularly in resource-limited regions like rural China, are often the first point of contact for patients but face significant challenges in providing comprehensive care for PCOS-IR. These facilities typically lack advanced diagnostic tools and laboratory capacity, making insulin testing or specialized biomarkers impractical due to high costs and the need for trained personnel. This diagnostic gap delays timely IR identification and intervention.

To address the limitations of existing studies, we developed a simple, practical nomogram for primary care settings. Our nomogram uses readily available clinical parameters obtained during routine checkups, unlike previous models that depend on insulin measurements or advanced biomarker. This model bridges the gap between complex diagnostic tools and practical use in resource-limited settings, offering an accessible method for early IR risk identification in patients with PCOS. By providing a practical and scalable tool, this nomogram supports more effective screening and management of IR in under-resourced settings.

## 2 Research methods

### 2.1 Study design and participants

This was a retrospective observational cohort study conducted in Jiangsu Province Hospital of Traditional Chinese Medicine. This type of design enables preliminary model development and validation using existing clinical data, while facilitating the collection of large-scale samples. It is suitable for exploring preliminary models for PCOS-IR risk prediction and laying the foundation for future prospective studies.

We included women aged 18–45 years with untreated PCOS-IR, diagnosed in the outpatient department of Jiangsu Province Hospital of Traditional Chinese Medicine between January 2023 and December 2023. PCOS diagnosis was based on the 2023 International Evidence-based Guidelines for the Assessment and Management of PCOS (13). The diagnosis requires the presence of two of the following conditions: (i) clinical/biochemical hyperandrogenism; (ii) ovulation dysfunction; and (iii) ultrasound showing polycystic ovaries, or anti-Mullerian hormone (AMH) instead of ultrasound. Specifically, according to the modified Ferriman Gallway score, clinical hyperandrogenism was diagnosed in Chinese women with a score of ≥4 or a score of ≥2 including the upper lip, lower abdomen, and inner thigh. Free testosterone index (FAI) = total testosterone (nmol/L)×100/sex hormone-binding globulin (nmol/L), and FAI > 6.4 was defined as biochemical hyperandrogenism. Ovulatory dysfunction was defined as less than 21 or more than 45 days after menarche; from 3 years after menarche to perimenopause: less than 21 or more than 35 days, or fewer than 8 cycles per year; more than 90 days for any cycle within 1 year after menarche; primary amenorrhea at 15 years of age or 3 years after breast development. PCOM is indicated by ultrasound, indicating a single/bilateral ovarian volume >10 mL, or the presence of 12 or more number of follicles 2–9 mm in diameter. Exclusion criteria included pre-existing conditions such as type 2 diabetes, cardiovascular diseases, thyroid disorders, Cushing’s syndrome, and any medications affecting glucose metabolism. This single-center design may introduce selection bias as it reflects the patient population of this specific institution. Future multi-center studies are planned to validate the model and reduce potential biases related to patient diversity.

This study was approved by the Ethics Committee of Nanjing University of Chinese Medicine (2022NL-187-02). This study is a retrospective analysis of routine clinical data; the Ethics Committee of Nanjing University of Chinese Medicine waived the necessity of informed consent.

### 2.2 Clinical data collection and the assessment of insulin resistance

The primary outcome of this study was the assessment of patients with PCOS and IR. During the study period, we recruited subjects with suspected PCOS-IR, as determined by clinicians at the time of visit based on serologic testing. Given the stronger linear correlation between HOMA-IR and HIEC (14), the diagnosis of IR in this study was based on the HOMA-IR index:


HOMA−IR = [fasting insulin (μIU/mL)]×[fasting glucose (mmol/L)]/22.5

A HOMA-IR value greater than 2.6 was considered indicative of IR (15), and these patients were classified as IR-positive. Patients diagnosed with PCOS but HOMA-IR ≤ 2.6 were classified as IR-negative.

Demographic data collected included age (in years), body mass index (BMI, calculated as weight in kilograms divided by height in meters squared) (16), family history of PCOS (defined as positive if the patient’s mother or grandmother had a history of PCOS), disease duration (time between PCOS diagnosis and the diagnosis of comorbid IR), and the longest recorded menstrual cycle length (UML, e.g., if a patient’s cycle length from 30 to 120 days, the UML recorded as 120 days) (9).

Laboratory measurements included fasting plasma glucose (FPG), AMH (17), testosterone (T) (18), sex hormone-binding globulin (SHBG) (19), total cholesterol (TC) (20), triglyceride (TG) (21), high-density lipoprotein (HDL) (22), low-density lipoprotein (LDL) (23), aspartate aminotransferase (AST) (24), alanine aminotransferase (ALT), and serum uric acid (SUA) (25). FPG was measured from blood samples taken after at least 8 h of fasting. AMH was measured using enzyme-linked immunosorbent assay (ELISA), T was determined by radioimmunoassay, and SHBG was measured using chemiluminescence immunoassay. TC was analyzed by automated enzymatic method, TG was analyzed by direct enzymatic colorimetry, and HDL was analyzed by direct enzymatic method. LDL was calculated using the Friedewald formula for patients with triglycerides<4.5 mmol/L. AST and ALT were measured using kinetic enzymatic methods, and SUA was measured using the uricase-peroxidase method.

The selection of potential predictors was based on factors identified in previous clinical studies as having prognostic value, as well as on factors considered clinically relevant by the investigators.

### 2.3 Statistical analysis

The sample size of this study was based on available data, and no prior power calculation was performed. We identified medical record data for 141 normal controls and 571 patients with PCOS. The internal validation set was a randomly selected portion of the training set used for model selection and parameter tuning. The external validation set, used as the test set, was chronologically separated from the main dataset to evaluate the generalizability of the nomogram in the PCOS population. Given the relatively small sample size (<10,000), a 7:3 data split ratio was used (26). A total of 171 patients (30% of the cohort) recruited after 1 September 2023 formed the external validation set, while the 400 patients recruited earlier were randomly split into an internal training set (280 patients) and an internal validation set (120 patients). Continuous variables were transformed into categorical variables to enhance clinical applicability. Independent variable groupings were based on the laboratory reference standards of our center and clinical experience.

Missing data were managed using multiple imputation based on fully conditional specification, ensuring unbiased estimates. Sensitivity analyses were performed using different imputation strategies to assess the robustness of the results (**Supplementary Table 1**). Descriptive statistics are presented as frequencies (percentages) for categorical variables and as means (SD) for continuous variables. One-way ANOVA was used for continuous variables, and the chi-square test was used for categorical variables to compare distributions across the three cohorts.

**Figure 1:**
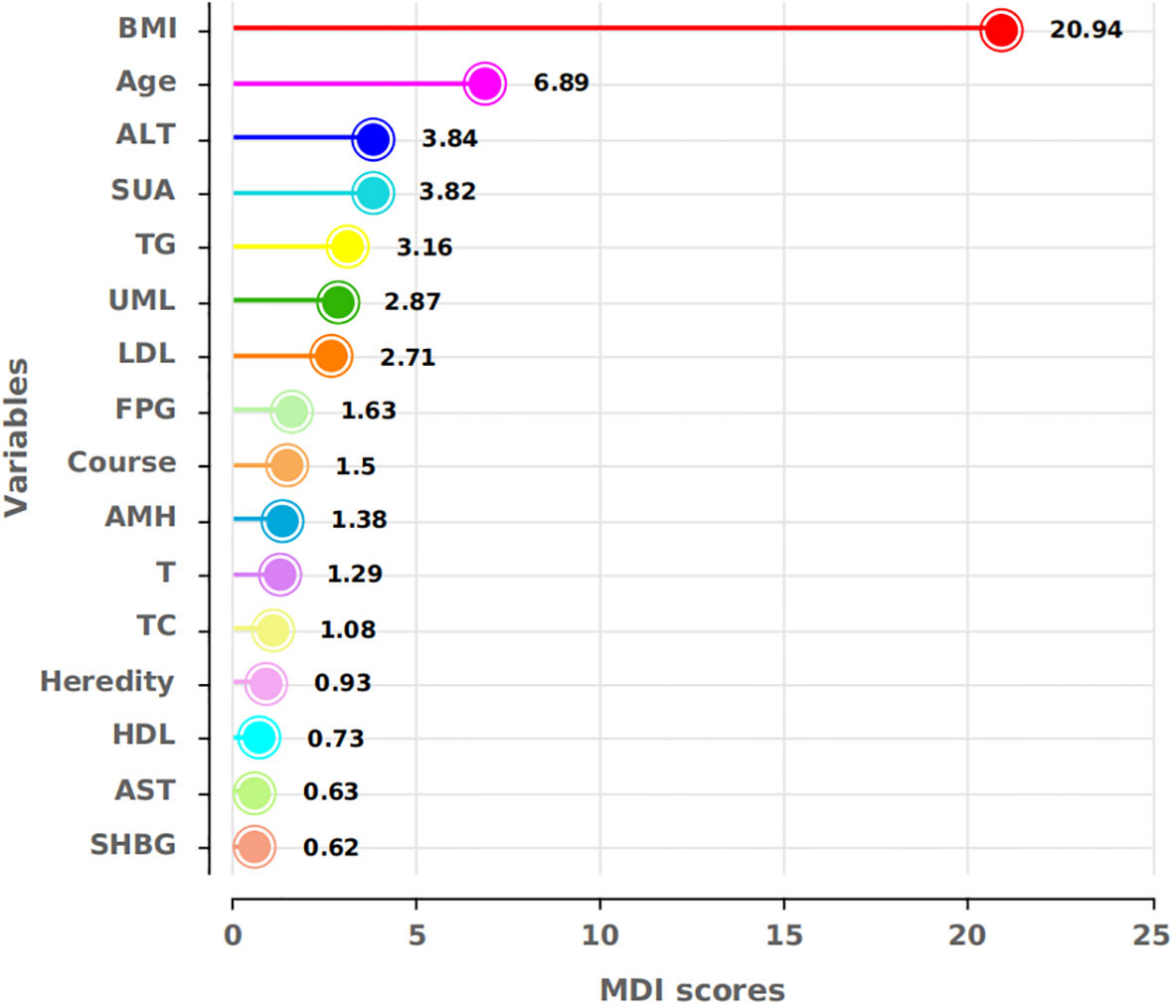
MDI scores for the respective variables. BMI, FPG, ALT, SUA, TG, and LDL showed high MDI scores in the RF, indicating their importance for model construction.

**Figure 2:**
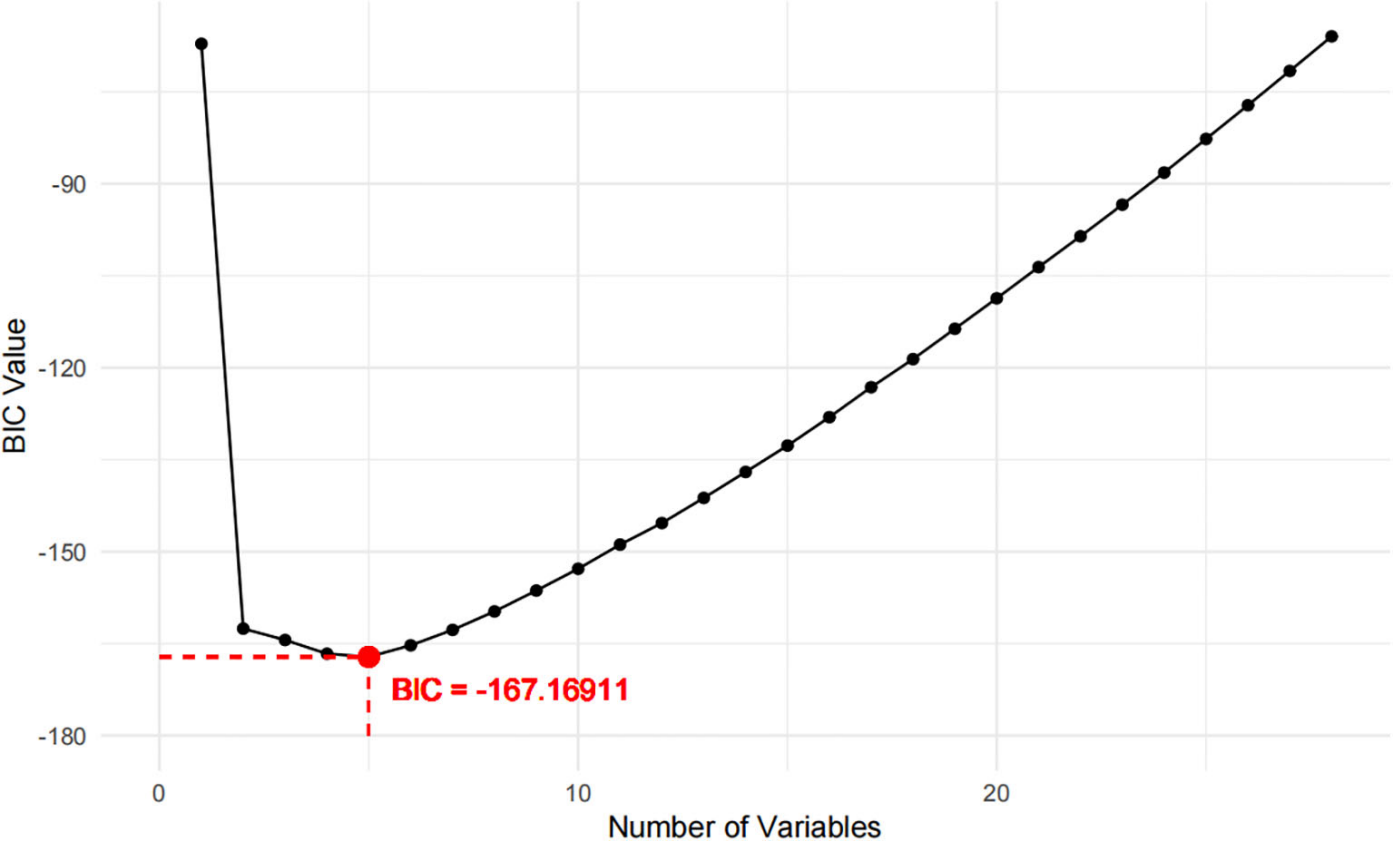
BIC values across different model sizes.

**Figure 3:**
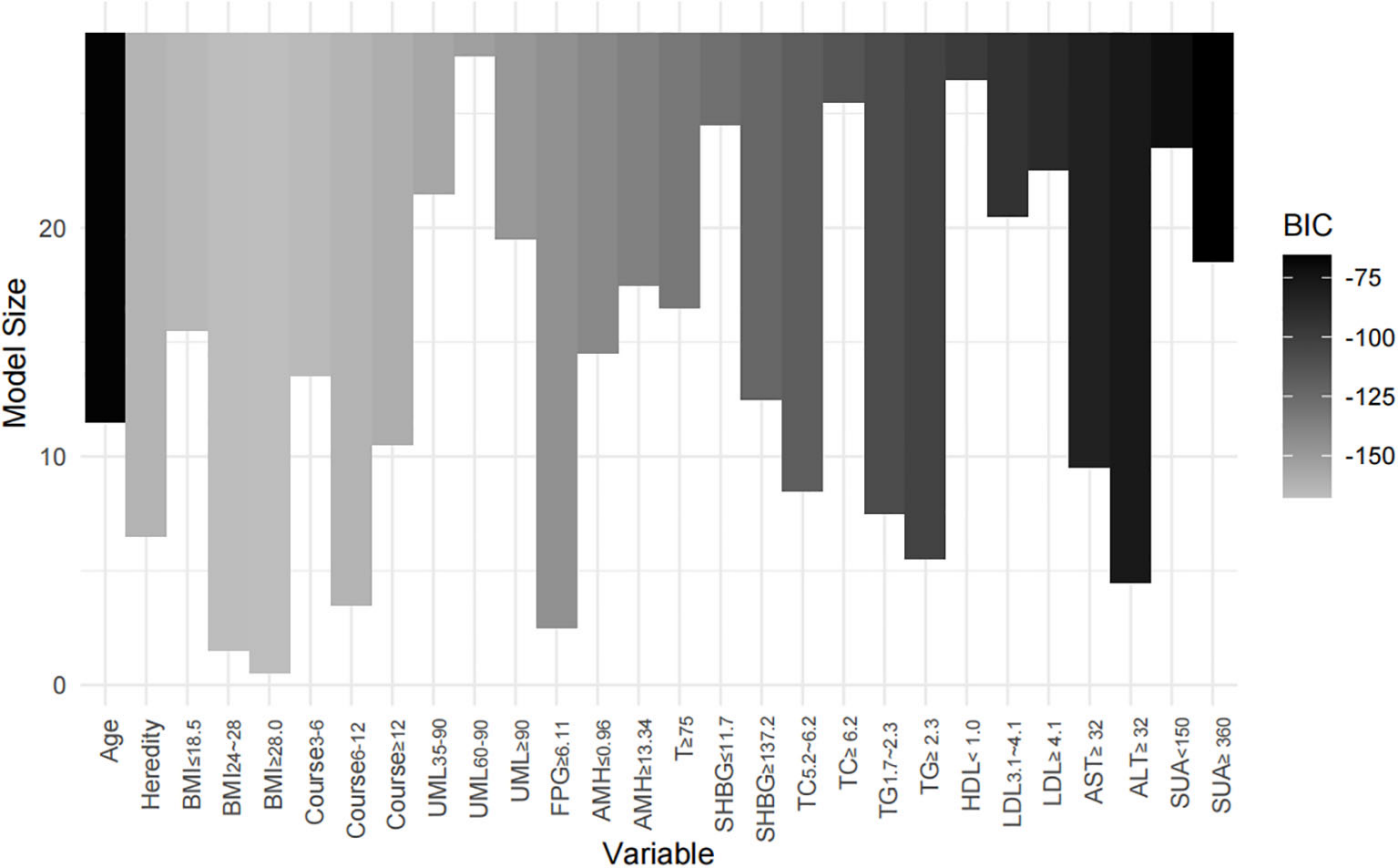
Impact of variable selection on BIC. The models containing BMI, course, FPG, and ALT achieved the lowest BIC values, indicating that this combination of variables provided the best model fit.

Two models were developed for predictor selection and risk estimation: Model 1 utilized logistic regression combined with the random forest (RF) algorithm, while Model 2 was based on the best subset regression (BSR) method using the Bayesian information criterion (BIC). In Model 1, the RF algorithm was used to screen independent variables. Cross-validation determined the optimal parameters, with ntree set to 200 and mtry set to 4, resulting in an out-of-bag (OOB) error rate of 12.74%, indicating optimal model performance (27). Mean decrease impurity (MDI) was used to rank variable importance (**Figure 1**). Through univariate analysis, 11 variables were found to be significantly associated with IR risk (**Table 1**). Six of these variables—BMI, FPG, ALT, SUA, TG, and LDL—showed significant results in univariate analysis (*p* < 0.05) and high MDI rankings (top 50%) in the RF analysis. These six variables were further included in multivariate logistic regression, and BMI, FPG, ALT, and TG were ultimately selected as the main predictors for Model 1. This combination of logistic regression and the RF algorithm was chosen to balance model interpretability and efficient feature selection, ensuring a robust prediction model for PCOS-IR risk stratification. For Model 2, we employed the BSR method to select the optimal combination of variables, using the BIC as the selection criterion (28). The lowest BIC value of −167.16911 was reached when it included five variables, marking the optimal inflection point (**Figure 2**). The BIC values for each variable across different model sizes are shown in **Figure 3**, where darker bars indicate lower BIC values, representing a better model fit. At the optimal BIC inflection point, the selected variables were BMI (24.0–28 and ≥28.0 kg/m^2^), disease course (6–12 months), FPG (≥6.11 mmol/L), and ALT (≥32 U/L). Since two BMI categories were included in the BSR method, they were treated as the same variable, and we ultimately included BMI, disease course, FPG, and ALT to construct Model 2.

**Table 1:** Results of logistic regression combined with RF for variable screening.

Variables	Level	Univariate logistic regression OR (95% CI) *p*-value		Multivariate logistic regression β S.E. OR (95% CI) *p*-value			
BMI (kg/m^2^)	18.5–24	Ref				Ref	
	≤18.5	0.94 [0.78, 1.13]	0.968	−15.27	13.73	0.97 [0.57, 1.64]	0.991
	24–28	4.80 [2.17, 8.59]	<0.001	2.53	0.44	2.60 [1.50, 3.47]	<0.001
	≥28	3.40 [2.41, 7.10]	<0.001	3.87	0.57	4.01 [1.70, 6.70]	<0.001
FPG (mmol/L)	3.89–6.11	Ref				Ref	
	≥6.11	1.47 [1.15, 1.88]	0.011	2.58	1.21	3.26 [1.76, 9.75]	0.032
T (ng/dL)	0–75	Ref					
	≥75	1.83 [1.08, 3.18]	0.027				
SHBG (nmol/L)	11.7–137.2	Ref					
	≤11.7	5.65[1.62, 8.34]	0.042				
	≥137.2	0.43 [0.06, 2.62]	0.357				
TC (mmol/L)	<5.2	Ref					
	5.2–6.2	2.49 [1.41, 4.57]	0.002				
	≥6.2	2.26 [0.94, 6.03]	0.082				
TG (mmol/L)	<1.7	Ref				Ref	
	1.7–2.3	2.12 [1.09, 4.31]	0.032	0.40	0.51	1.49 [0.56, 4.17]	0.437
	≥2.3	2.15 [3.26, 6.50]	<0.001	1.33	0.68	3.80 [1.09, 6.45]	0.049
HDL (mmol/L)	<1.0	0.27 [0.09, 0.67]	0.009				
	≥1.0	Ref					
LDL (mmol/L)	<3.1	Ref				Ref	
	3.1–4.1	3.09 [1.74, 5.69]	<0.001	0.69	0.43	1.99 [0.87, 4.77]	0.112
	≥4.1	3.84 [1.84, 6.78]	0.013	1.21	1.16	3.34 [0.41, 8.92]	0.301
AST (U/L)	<32	Ref					
	≥32	1.46 [1.13, 1.88]	0.009				
ALT (U/L)	<32	Ref				Ref	
	≥32	1.08 [1.02, 1.43]	<0.001	1.11	0.49	3.03 [1.20, 8.49]	0.025
SUA (μmol/L)	150–360	Ref				Ref	
	<150	0.85 [0.61, 1.17]	0.981	15.14	27.44	3.46 [0.58, 1.69]	0.996
	≥360	4.59 [2.72, 7.92]	<0.001	0.52	0.39	1.69 [0.79, 3.59]	0.176

BMI, body mass index (kg/m²); FPG, fasting plasma glucose (mmol/L); T, testosterone (ng/dL); SHBG, sex hormone-binding globulin (nmol/L); TC, total cholesterol (mmol/L); TG, triglycerides (mmol/L); HDL, high-density lipoprotein (mmol/L); LDL, low-density lipoprotein (mmol/L); AST, aspartate aminotransferase (U/L); ALT, alanine aminotransferase (U/L); SUA, serum uric acid (μmol/L).

Values are expressed as number (%), unless indicated otherwise.

Univariate regression identified 11 variables significantly associated with IR risk. RF was used to select six variables (BMI, FPG, ALT, SUA, TG, and LDL) for multivariate regression, and BMI, FPG, ALT, and TG were identified as the main predictors in Model 1.

**Figure 4:**
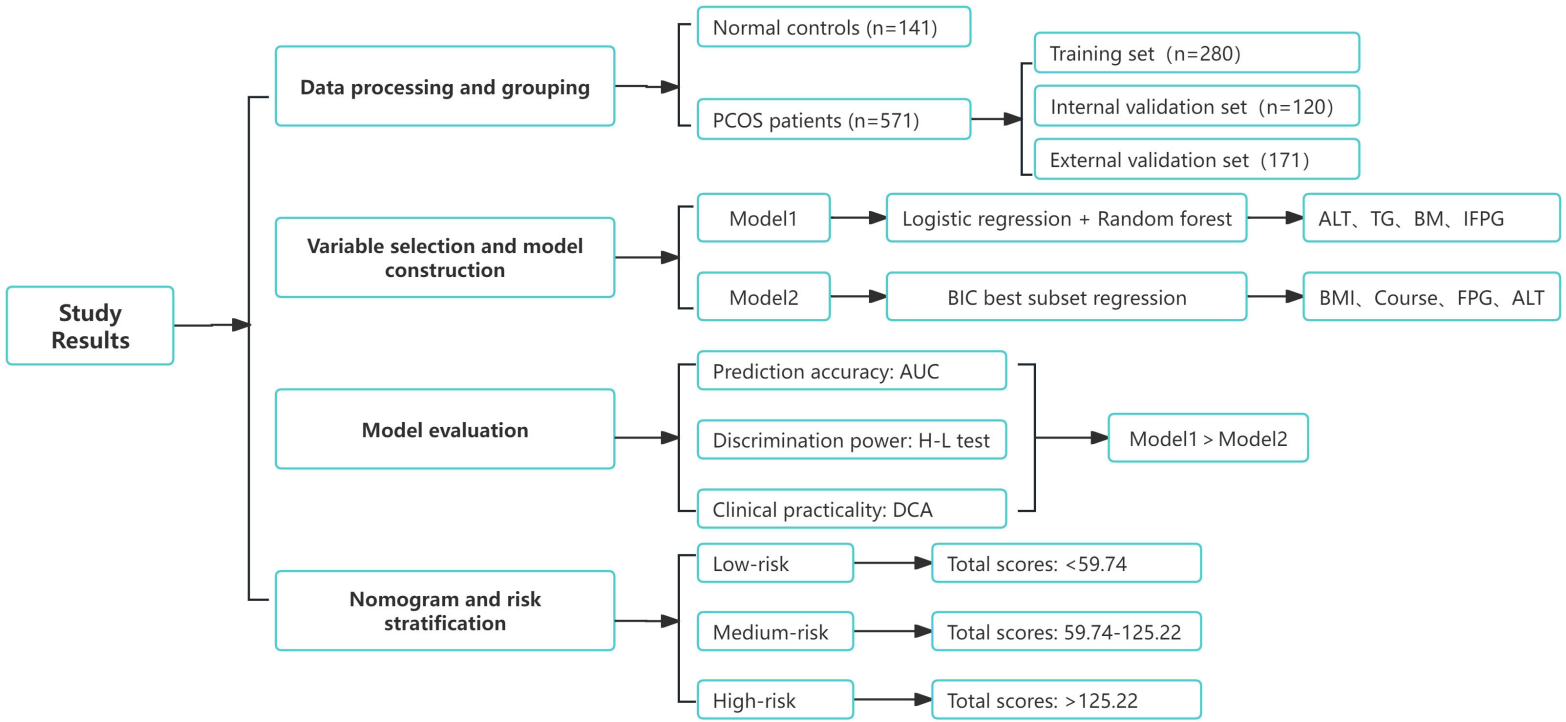
Flowchart of study results. The complete process from data processing and grouping to model selection, evaluation, and risk stratification is shown.

The performance of both models was evaluated in all three sets (training, internal validation, and external validation) using the area under the ROC curve (AUC), calibration curve, and decision curve analysis (DCA). The final nomogram was derived from the best-performing model, assigning a total score to each patient based on the combined predictors. The cut function was used for risk stratification based on individual total scores. All analyses were conducted using R version 3.6.1, with statistical significance set at *p* < 0.05 (two-tailed). This study adheres to the TRIPOD guidelines (29) for transparent reporting of multivariable prediction models for individual prognosis or diagnosis (**Supplementary Material**). **Figure 4** illustrates the complete flowchart of the study methods.

## 3 Results

### 3.1 Demographic and clinical characteristics

Data from 141 normal controls and 571 patients with PCOS were analyzed. Most predictor variables, except for HDL, showed statistically significant differences between the PCOS group and the normal controls (*p* < 0.05), highlighting the predictive strength of the selected variables (**Table 2**).

**Table 2:** Characteristics of patients with PCOS (*N* = 571) and normal controls (*N* = 141).

Variables	PCOS	Normal	*p*-value
No.	571	141	
Age (years) (SD)	26.62 (5.11)	30.23 (7.35)	0.040^#^
Family history of PCOS			
No	465 (81.4)	128 (90.8)	0.011^*^
Yes	106 (18.6)	13 (9.2)	
Course (months)			
≤3	2 (0.4)	NA	NA
3–6	8 (1.4)	NA	
6–12	76 (13.3)	NA	
≥12	485 (84.9)	NA	
BMI (kg/m^2^)			
18.5–24	166 (29.1)	84 (59.6)	<0.001^*^
≤18.5	15 (2.6)	18 (12.8)	
24–28	188 (32.9)	30 (21.3)	
≥28	202 (35.4)	9 (6.4)	
UML (days)			
≤35	59 (10.3)	85 (60.3)	<0.001^*^
35–60	84 (14.7)	41 (29.1)	
60–90	88 (15.4)	9 (6.4)	
≥90	340 (59.6)	6 (4.3)	
FPG (mmol/L)			
3.89–6.11	528 (92.5)	139 (98.6)	0.013^*^
≥6.11	43 (7.5)	2 (1.4)	
AMH (nmol/L)			
0.96–13.34	436 (76.4)	110 (78.0)	<0.001^*^
≤0.96	4 (0.7)	26 (18.4)	
≥13.34	131 (22.9)	5 (3.5)	
T (ng/dL)			
0–75	386 (67.6)	127 (90.1)	<0.001^*^
≥75	185 (32.4)	14 (9.9)	
SHBG (nmol/L)			
11.7–137.2	532 (93.2)	137 (97.2)	<0.001^*^
≤11.7	30 (5.3)	1 (0.7)	
≥137.2	9 (1.5)	3 (2.1)	
TC (mmol/L)			
<5.2	343 (60.1)	113 (80.1)	<0.001^*^
5.2–6.2	164 (28.7)	23 (16.3)	
≥6.2	64 (11.2)	5 (3.5)	
TG (mmol/L)			
<1.7	391 (68.5)	132 (93.6)	<0.001^*^
1.7–2.3	96 (16.8)	9 (6.4)	
≥2.3	84 (14.7)	0 (0.0)	
HDL (mmol/L)			
<1.0	59 (10.3)	8 (5.7)	0.125^*^
≥1.0	512 (89.7)	133 (94.3)	
LDL (mmol/L)			
<3.1	362 (63.4)	115 (81.6)	<0.001^*^
3.1–4.1	173 (30.3)	24 (17.0)	
≥4.1	36 (6.3)	2 (1.4)	
AST (U/L)			
<32	502 (87.9)	133 (94.3)	0.041^*^
≥32	69 (12.1)	8 (5.7)	
ALT (U/L)			
<32	410 (71.8)	129 (91.5)	<0.001^*^
≥32	161 (28.2)	12 (8.5)	
SUA (μmol/L)			
150–360	303 (53.1)	124 (87.9)	<0.001^*^
<150	3 (0.5)	2 (1.4)	
≥360	265 (46.4)	15 (10.6)	

BMI, body mass index (kg/m²); UML, upper menstrual cycle limit (days); FPG, fasting plasma glucose (mmol/L); AMH, anti-Mullerian hormone (nmol/L); T, testosterone (ng/dL); SHBG, sex hormone-binding globulin (nmol/L); TC, total cholesterol (mmol/L); TG, triglycerides (mmol/L); HDL, high-density lipoprotein (mmol/L); LDL, low-density lipoprotein (mmol/L); AST, aspartate aminotransferase (U/L); ALT, alanine aminotransferase (U/L); SUA, serum uric acid (μmol/L).

Values are expressed as number (%), unless otherwise indicated.

^#^ Independent *t*-test.

^*^Chi-square test.

NA, Not Applicable.

Comparisons across the training, internal validation, and external validation sets revealed no statistically significant differences in demographic or clinical characteristics (*p* > 0.05) (**Table 3**), ensuring internal consistency for model construction and validation.

**Table 3:** Characteristics of the patients in the training and validation sets (*N* = 571).

Variables	Overall	Training set	Internal validation set	External validation set	*p*-value
No. of patients	571	280	120	171	
Age (years) (SD)	26.62 (5.11)	26.51 (5.03)	26.89 (5.29)	26.70 (5.13)	0.301^#^
Family history of PCOS					
No	465 (81.4)	229 (81.8)	97 (80.8)	139 (81.3)	0.933^*^
Yes	106 (18.6)	51 (18.2)	23 (19.2)	32 (18.7)	
Course (months)					
≤3	2 (0.4)	0 (0.0)	1 (0.8)	1 (0.6)	0.397^*^
3–6	8 (1.4)	5 (1.8)	1 (0.8)	2 (1.2)	
6–12	76 (13.3)	36 (12.9)	17 (14.2)	23 (13.4)	
≥12	485 (84.9)	239 (85.3)	101 (84.2)	145 (84.8)	
BMI (kg/m^2^)					
18.5–24	166 (29.1)	77 (27.5)	38 (31.6)	51 (29.8)	0.504^*^
≤18.5	15 (2.6)	9 (3.2)	2 (1.7)	4 (2.3)	
24–28	188 (32.9)	98 (35.0)	35 (29.2)	55 (32.2)	
≥28	202 (35.4)	96 (34.3)	45 (37.5)	61 (35.7)	
UML (days)					
≤35	59 (10.3)	29 (10.4)	12 (10.0)	18 (10.5)	0.885^*^
35–60	84 (14.7)	38 (13.6)	20 (16.7)	26 (15.2)	
60–90	88 (15.4)	44 (15.7)	18 (15.0)	26 (15.2)	
≥90	340 (59.6)	169 (60.3)	70 (58.3)	101 (59.1)	
FPG (mmol/L)					
3.89–6.11	528 (92.5)	255 (91.1)	114 (95.0)	159 (92.9)	0.253^*^
≥6.11	43 (7.5)	25 (8.9)	6 (5.0)	12 (7.1)	
AMH (nmol/L)					
0.96–13.34	436 (76.4)	221 (78.9)	86 (71.7)	129 (75.4)	0.287^*^
≤0.96	4 (0.7)	2 (0.7)	1 (0.8)	1 (0.6)	
≥13.34	131 (22.9)	57 (20.4)	33 (27.5)	41 (24.0)	
T (ng/dL)					
0–75	386 (67.6)	184 (65.7)	85 (70.8)	117 (68.4)	0.377^*^
≥75	185 (32.4)	96 (34.3)	35 (29.2)	54 (31.6)	
SHBG (nmol/L)					
11.7–137.2	532 (93.2)	258 (92.1)	114 (95.0)	160 (93.6)	0.134^*^
≤11.7	30 (5.3)	19 (6.8)	3 (2.5)	8 (4.7)	
≥137.2	9 (1.5)	3 (1.1)	3 (2.5)	3 (1.7)	
TC (mmol/L)					
<5.2	343 (60.1)	171 (61.1)	70 (58.3)	102 (59.6)	0.090^*^
5.2–6.2	164 (28.7)	85 (30.3)	31 (25.8)	48 (28.1)	
≥6.2	64 (11.2)	24 (8.6)	19 (15.9)	21 (12.3)	
TG (mmol/L)					
<1.7	391 (68.5)	192 (68.6)	82 (68.3)	117 (68.4)	0.483^*^
1.7–2.3	96 (16.8)	43 (15.3)	23 (19.2)	30 (17.5)	
≥2.3	84 (14.7)	45 (16.1)	15 (12.5)	24 (14.1)	
HDL (mmol/L)					
<1.0	59 (10.3)	27 (9.6)	14 (11.7)	18 (10.5)	0.666^*^
≥1.0	512 (89.7)	253 (90.4)	106 (88.3)	153 (89.5)	
LDL (mmol/L)					
<3.1	362 (63.4)	187 (66.8)	69 (57.5)	106 (62.0)	0.140^*^
3.1–4.1	173 (30.3)	75 (26.8)	44 (36.7)	54 (31.6)	
≥4.1	36 (6.3)	18 (6.4)	7 (5.8)	11 (6.4)	
AST (U/L)					
<32	502 (87.9)	244 (87.1)	107 (89.2)	151 (88.3)	0.690^*^
≥32	69 (12.1)	36 (12.9)	13 (10.8)	20 (11.7)	
ALT (U/L)					
<32	410 (71.8)	204 (72.9)	84 (70.0)	122 (71.3)	0.644^*^
≥32	161 (28.2)	76 (27.1)	36 (30.0)	49 (28.7)	
SUA (μmol/L)					
150-360	303 (53.1)	151 (53.9)	62 (51.7)	90 (52.6)	0.577^*^
<150	3 (0.5)	2 (0.7)	0 (0.0)	1 (0.6)	
≥360	265 (46.4)	127 (45.4)	58 (48.3)	80 (46.8)	

BMI, body mass index (kg/m²); UML, upper menstrual cycle limit (days); FPG, fasting plasma glucose (mmol/L); AMH, anti-Mullerian hormone (nmol/L); T, testosterone (ng/dL); SHBG, sex hormone-binding globulin (nmol/L); TC, total cholesterol (mmol/L); TG, triglycerides (mmol/L); HDL, high-density lipoprotein (mmol/L); LDL, low-density lipoprotein (mmol/L); AST, aspartate aminotransferase (U/L); ALT, alanine aminotransferase (U/L); SUA, serum uric acid (μmol/L).

Values are expressed as number (%), unless indicated otherwise.

^#^ One-way ANOVA test.

^*^Chi-square test.

There were no significant differences in demographic and clinical characteristics among the three sets.

### 3.2 Screening of predictor variables

For Model 1, univariate analysis and RF screening results were combined. Six variables—BMI, FPG, ALT, SUA, TG, and LDL—were included in the multivariate logistic regression analysis (**Table 1**). After this analysis, BMI, FPG, ALT, and TG were identified as the most significant predictors (*p* < 0.05).

For Model 2, the BSR method based on BIC selected four variables: BMI (categorized as 24.0–28 and ≥28.0 kg/m²), disease course (6–12 months), FPG (≥6.11 mmol/L), and ALT (≥32 U/L). This model achieved the lowest BIC value (−167.16911) (**Figure 2**), with the BIC values across model sizes shown in **Figure 3**.

### 3.3 Comparison of model evaluation

**Figure 5:**
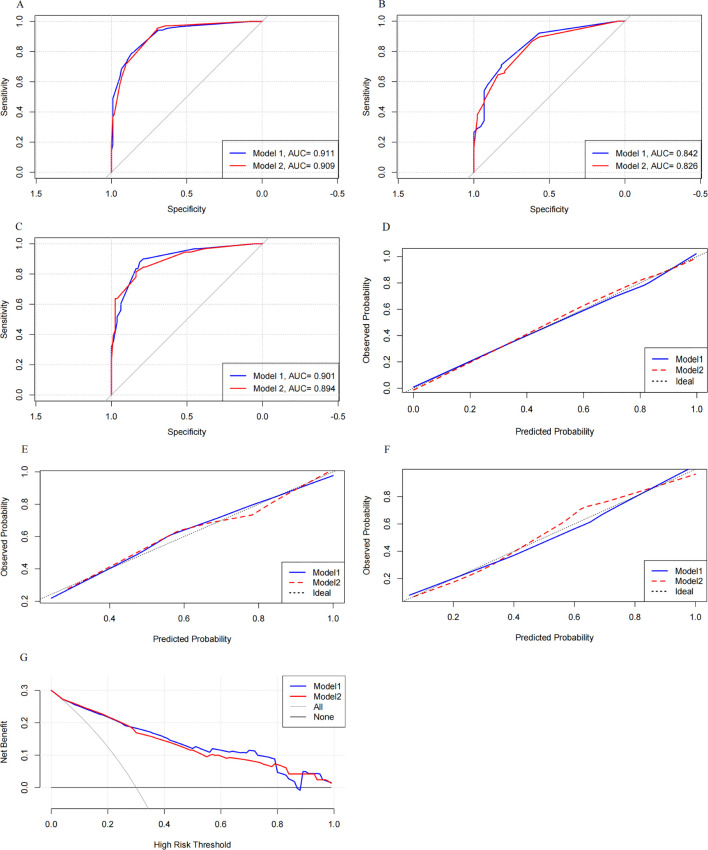
Comparison of the discrimination, calibration, and clinical utility of the two models across the three sets. Model 1 showed higher AUC values than Model 2 in the training, internal validation, and external validation sets. It also demonstrated better calibration and provided greater net clinical benefit in the intermediate-risk range. ROC curves for the training set **(A)** and internal **(B)** and external **(C)** validation sets. Calibration curves with 1,000 bootstraps resamples for the training set **(D)** and internal **(E)** and external **(F)** validation sets. **(G)** DCA curves for both models.

We compared the performance of Model 1 and Model 2 across the training and validation sets (**Figures 5A–C**). Model 1 consistently achieved higher AUC values: 0.911 (95% CI 0.878–0.911) in the training set, 0.842 (95% CI 0.771–0.842) in the internal validation set, and 0.901 (95% CI 0.856–0.901) in the external validation set. In comparison, Model 2 had slightly lower AUCs: 0.909 (95% CI 0.875–0.909), 0.826 (95% CI 0.754–0.826), and 0.894 (95% CI 0.848–0.894) in the respective datasets.

Calibration curves for both models were plotted (**Figures 5D–F**). Model 1’s curves were closer to the ideal diagonal line across all datasets, indicating better calibration. The Hosmer–Lemeshow (H-L) test showed that Model 1 had *p*-values of 0.985 in the training set, 0.741 in the internal validation set, and 0.826 in the external validation set, compared to Model 2’s *p*-values of 0.684, 0.683, and 0.687. These results suggest that both models were well-calibrated, with Model 1 performing slightly better.

DCA was used to access the clinical utility of both models (**Figure 5G**). Both models demonstrated higher clinical utility than the all-treatment and no-treatment strategies. Notably, Model 1 outperformed Model 2 in the intermediate-risk range (0.28 to 0.79), which is the typical risk range for PCOS-IR. This advantage likely stems from Model 1’s ability to capture complex interactions between predictors, providing more accurate risk prediction and greater net benefit for managing moderate-risk patients.

### 3.4 Nomogram establishment and risk stratification

**Figure 6:**
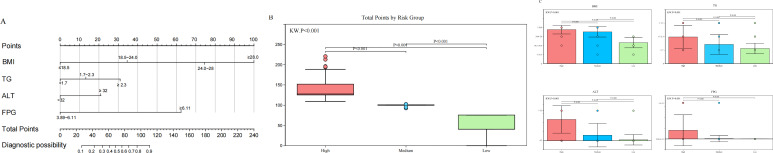
BMI, TG, ALT, and FPG, as independent predictors, effectively classify patients into low-, medium-, and high-risk groups, aiding clinical decision-making. **(A)** Predictive nomogram for PCOS-IR, incorporating BMI, TG, ALT, and FPG. **(B)** Distribution of total scores across risk groups. **(C)** Distribution of predictors stratified by risk groups.

After evaluating prediction accuracy, discrimination, and clinical utility, Model 1 was selected as the optimal model. A nomogram was developed using BMI (OR 1.43, 95% CI: 1.33–1.54), TG (OR 1.22, 95% CI: 1.02–1.52), ALT (OR 1.03, 95% CI: 1.01–1.04), and FPG (OR 5.19, 95% CI: 2.98–9.04) to predict PCOS-IR risk (**Figure 6A**).

Each patient’s risk score was calculated using the nomogram, with higher scores indicating greater risk. Patients were stratified into low-risk (≤59.74, *n* = 262), medium-risk (59.74–125.22, *n* = 130), and high-risk (>125.22, *n* = 179) groups based on total scores (**Figure 6B**). Significant differences in predictor distributions were observed between these groups (**Figure 6C**). The medium-risk group had a 9-fold higher likelihood of IR (OR = 9.14, 95% CI: 5.66–15.14), while the high-risk group had a 58-fold higher risk (OR = 58.63, 95% CI: 29.09–75.46), all with statistical significance (*p* < 0.001).

To illustrate the nomogram’s clinical application, consider a 35-year-old woman with PCOS, a BMI of 28 kg/m², a TG level of 2.2 mmol/L, an ALT level of 35 U/L, and an FPG level of 5.8 mmol/L. Her total score of 85.2 places her in the intermediate-risk group, indicating a moderate IR risk and suggesting the need for early intervention, such as lifestyle changes or medications to reduce future metabolic complications.

## 4 Discussion

Patients with PCOS exhibit a high prevalence of IR, which is strongly linked to metabolic and reproductive dysfunctions (30). Early identification of IR risk is crucial for preventing long-term complications. Current predictive models for IR often rely on insulin measurements, which are costly and not routinely performed. Our model, based on easily accessible clinical parameters such as BMI, TG, ALT, and FPG, offers a cost-effective tool for early identification of IR, particularly in primary care or resource-limited environments. By incorporating these routinely measured variables, our nomogram facilitates early interventions, eliminating the need for insulin testing. This approach provides a simpler and more accessible method for risk stratification and timely management of metabolic complications in patients with PCOS.

Several key markers emerged as strong predictors of IR. BMI was a significant independent predictor of IR, consistent with previous studies (31). Overweight and obesity were strongly associated with increased IR risk, likely due to the pro-inflammatory and lipotoxic effects of excess adipose tissue (32). Additionally, central obesity, common in patients with PCOS, impairs glucose metabolism and exacerbates IR (33). Obesity in PCOS has been linked to increased serine phosphorylation of insulin receptor substrate-1 (IRS-1), which further disrupts insulin signaling (34). These findings highlight the importance of weight management in reducing IR risk, particularly through lifestyle interventions aimed at promoting weight loss and improving insulin sensitivity in women with PCOS.

FPG and TG also emerged as independent predictors of IR. Elevated TG levels (≥1.7 mmol/L) were associated with an increased risk of IR, and this risk was further heightened in patients with TG levels ≥2.3 mmol/L. Similarly, elevated FPG (≥6.11 mmol/L) was strongly associated with IR risk. The combination of TG and FPG, as evidenced by the TyG index, is an effective predictor of IR (11, 35). Dyslipidemia and impaired glucose metabolism are common in PCOS and are exacerbated by obesity (36). The liver plays a critical role in insulin clearance and glucose metabolism. Dysregulated lipid metabolism in the liver contributes to hepatic IR, which exacerbates the metabolic profile of patients with PCOS (37).

In this study, ALT was identified as a novel independent predictor of IR, highlighting its role in early metabolic dysfunction in PCOS. Traditionally used as a marker of liver function, elevated ALT levels have been linked to hepatic IR, particularly in conditions like non-alcoholic fatty liver disease (NAFLD) (38). In this study, even mild elevations in ALT (≥32 U/L) were associated with higher IR risk, suggesting that ALT may signal early hepatic involvement in metabolic abnormalities. This aligns with studies linking ALT to visceral fat accumulation and hepatic IR (24, 37). Importantly, even patients with ALT levels in the upper normal range were found to have an increased risk of IR. This suggests that ALT could serve as an early indicator of IR, even before it exceeds normal limits, providing clinicians with an additional tool for assessing risk in patients with PCOS.

This study provides a clinically useful nomogram that allows for personalized risk stratification in patients with PCOS. By classifying patients into low-, medium-, and high-risk groups, clinicians can tailor interventions based on individual risk profiles. For low-risk patients, lifestyle modifications focusing on weight control and lipid management may be sufficient to prevent progression to IR. Medium-risk patients may require more intensive interventions, including pharmacological treatments, to mitigate their risk. High-risk patients, characterized by elevated BMI, TG, FPG, and ALT, are at the greatest risk and may benefit from aggressive management strategies, including lifestyle changes and medical therapies (39). This approach allows for early intervention, potentially improving metabolic and reproductive outcomes in patients with PCOS.

In conclusion, this study addresses key limitations of existing IR prediction models by offering a practical, accessible tool that does not rely on insulin testing. The nomogram provides a cost-effective means of identifying patients with PCOS at risk for IR, facilitating timely interventions that may mitigate long-term metabolic risks. However, this tool should complement clinical judgment, while offering valuable insights. Further validation through multi-center studies is crucial to confirm its broader applicability and refine its predictive accuracy.

## 5 Study limitations

This study has several limitations. First, the single-center design and retrospective nature may limit the generalizability of our findings. Data were collected from only one medical center, which restricts broader applicability. Additionally, retrospective data may introduce incomplete or inconsistent information, affecting the model’s stability and accuracy. Prospective, multi-center studies with larger and more diverse samples are necessary to validate the model’s performance, determine evidence-based treatment paradigms for each risk stratification, and ensure its practical utility across different clinical settings.

Second, variables influencing IR, such as vitamin D and thyroid function, were excluded due to missing data. A prospective study that includes these factors is underway, which we believe will enhance the model’s comprehensiveness and accuracy.

Third, PCOS-IR is a multifactorial condition influenced by factors such as ethnicity, diet, BMI, and genetics. As our study focused on Chinese women, the model’s predictive accuracy should be cautiously interpreted in other ethnic groups. Moreover, because of the heterogeneity of PCOS phenotypes, we did not perform subgroup analyses for different phenotypes in this study. Future research should focus on specific PCOS phenotypes to better understand the risk differences and mechanisms underlying the development of IR, which could lead to more personalized management strategies for patients.

## 6 Conclusions

The nomogram developed in this study offers a novel approach to assessing the risk of PCOS-IR, compared to existing risk stratification models that rely on insulin testing. By utilizing readily available clinical parameters—BMI, TG, ALT, and FPG—this tool provides a cost-effective and accessible method for the early detection of IR, making it especially suitable for resource-limited primary care settings.

This nomogram holds significant potential for improving clinical outcomes in patients with PCOS by facilitating timely, individualized interventions. Further prospective validation in diverse populations is necessary to ensure its broad applicability and enhance its impact in various healthcare environments.

## Data Availability

The original contributions presented in the study are included in the article/**Supplementary Material**. Further inquiries can be directed to the corresponding authors.
