# Supplementary material for: A nomogram to predict the risk of insulin resistance in Chinese women with polycystic ovary syndrome
Source: Front Endocrinol (Lausanne). 2024 Nov 27;15:1446827. doi: 10.3389/fendo.2024.1446827 (PMC11631621; doi:10.3389/fendo.2024.1446827)
Supplement: Supplementary file 1 [file DataSheet1.docx]

**TRIPOD Checklist:**

| **Title and abstract** | |
| --- | --- |
| Title | A nomogram to predict the risk of insulin resistance in Chinese women with polycystic ovary syndrome |
| Abstract | **OBJECTIVE:** In this study, the authors aimed to create a column-line diagram to accurately predict the risk of combined IR in patients with PCOS. **METHODS:** Patients with untreated PCOS-IR diagnosed in a single-center retrospective cohort study from January 2023 to December 2023 were enrolled in the present study and used for the construction and validation of the column-line diagrams.The area under the ROC curve (AUC) was used for the modeling of the model, calibration curve and Hosmer-Lemeshow goodness-of-fit tests, and decision curve analysis (DCA) were used to assess the model's discrimination, calibration, and clinical decision-making performance. Then, a risk stratification model based on nomograms was developed. **RESULTS:** A total of 571 patients were enrolled in the study; 400 patients enrolled before September 2023 were divided into training and validation sets, and 171 subsequently enrolled patients were used as the external validation set. Variables including BMI (OR 1.43), TG (OR 1.22), ALT (OR 1.03), and FPG (OR 5.19) were screened and included by logistic regression combined with a random forest algorithm to develop column line plots. The AUCs were 0.911 (95% CI 0.878-0.911), 0.842 (95% CI 0.771-0.842), 0.901 (95% CI 0.856-0.901) in the training, internal validation, and external validation sets, respectively. According to the calibration curve and HL test, the nomogram performed well in terms of agreement between predictions and actual observations. Using the final score of this column-line plot, patients could be categorized into three different (low, medium, and high) risk groups. **CONCLUSIONS:** Independent predictors of untreated PCOS-IR risk were selected to create a nomogram for diagnostic prediction of IR risk. Normograms can categorize patients into different risk groups and help in clinical decision making. |
| **Introduction** | |
| Background and objectives | Polycystic ovary syndrome (PCOS) is the most common endocrine and metabolic disorder among women of childbearing age, affecting the entire life cycle with a prevalence of 5-18%. Approximately 44% to 70% of patients with PCOS have significant comorbid IR, and obese patients have a higher risk of comorbid IR.IR is considered a major driver of PCOS pathophysiology.Women with PCOS comorbid IR (PCOS-IR) are at a significantly increased risk of adverse pregnancy outcomes and chronic diseases such as cardiovascular disease, type 2 diabetes mellitus, and metabolic syndrome, with significant health and economic costs. Therefore, early detection of the risk of combined IR is critical for women with PCOS.  The high insulin-normal glucose clamp (HIEC) method is still considered the gold standard for the assessment of IR, a test that requires special equipment and specialized technicians, is both expensive and time-consuming, and is currently used only in scientific studies. In large-scale epidemiological studies, simple and easy-to-use alternative indices are mainly chosen, such as homeostasis model assessment of insulin resistance (HOMA-IR), quantitative insulin sensitivity check index (QISCI), and the HIEC (HIEC). quantitative insulin sensitivity check index (QUICKI), etc. However, there is no standardized cut-off value for these assessment indexes.  The increasing incidence and complex clinical presentation of PCOS-IR emphasize the need for improved disease recognition in the clinical setting. Currently, some predictive models have been developed to identify risk factors for PCOS-IR. Notably, the onset of PCOS-IR may vary by race, geographic diet, BMI, and other factors. Therefore, in this study, we aimed to develop a diagnostic model for predicting the risk of comorbid IR for Chinese women with PCOS and to propose a risk stratification strategy based on nomograms for clinical decision-making. |
| **Methods** | |
| Source of data and participants | This was a retrospective observational cohort study conducted in Jiangsu Province Hospital of Traditional Chinese Medicine. We screened between January 2023 and December 2023 in jiangsu province, Chinese medicine hospital outpatient diagnosis of untreated patients with PCOS with IR and incorporated into our obserational cohort. |
| Outcome | The primary endpoint of this study was to evaluate PCOS patients with comorbid IR. During the study period, we recruited subjects with suspected PCOS-IR, which was defined by clinicians at the time of presentation after serologic testing of such patients。 |
| Predictors | Demographic data collected included age, body mass index (BMI), family history of PCOS, disease duration, and the maximum duration of the menstrual cycle (UML). Laboratory measurements included fasting glucose (FPG), anti-Müllerian hormone (AMH), testosterone (T), sex hormone-binding globulin (SHBG), total cholesterol (TC), triglycerides (TG), high-density lipoproteins (HDL), low-density lipoproteins (LDL), aminotransferase (AST), alanine aminotransferase (ALT), and serum uric acid (SUA). |
| Sample size | The sample size for this study was derived from the available data and there was no pre-calculation of efficacy for the sample size. Our final screening identified medical record data from 571 patients with PCOS. |
| Missing data | In this study, we use Multiple Imputation (MIM) to deal with the problem of missing values in the dataset. First, we interpolate the missing data using Gibbs sampling method. In order to ensure the stability of the interpolation results, we generate five different complete datasets. Specifically, for numeric variables, we used Predictive Mean Matching (PMM) method for interpolation; for binary variables, we used Logistic Regression model for interpolation. After data interpolation was completed, we performed Logistic Multifactor Regression analysis for each complete data set. We found that the regression coefficients and significance levels of the interpolated datasets were essentially the same, indicating that the missing value filling method used was reliable.(Appendix Table 1.) |
| Statistical analysis methods | We split the dataset using a 7:3 data split ratio. We randomly selected 171 patients (30% of the total cohort) to constitute the external validation set, with September 1, 2023 as the cut-off point. The 400 patients recruited before September 2023 were randomized into an internal training set (280) and an internal validation set (120).  For the screening of potential predictor variables, we used two methods for selecting the best combination of variables to construct 2 models respectively - model 1 based on traditional logistic regression combined with the random forest algorithm (RF); and model 2 based on the best subset regression (BSR) method determined by the Bayesian information criterion (BIC) .  Area under the ROC curve (AUC), calibration curve and Hosmer-Lemeshow goodness-of-fit tests, and decision curve analysis (DCA) were used to assess the discriminatory, calibration, and clinical decision-making performance of the 2 models. |
| Risk groups | After comparing predictive accuracy, discrimination, and clinical utility, we finally determined that Model 1 was the best model. Nomograms were constructed based on the predictor variables screened by this model (containing BMI, TG, ALT, and FPG). Each patient was given a total score calculated from the points obtained from the combined predictors, and risk stratification was performed using the cut function for individual total score distribution characteristics. |
| Development vs. validation | No statistically significant differences (p < 0.05) were observed in demographic and clinical characteristics of the patients in the three cohorts (training cohort and internal and external validation cohorts) when compared between groups. Specific data are shown in Table 1. |
| **Results** | |
| Participants | The characteristics of the participants (basic demographics, clinical features, available predictors) are shown in Table 1. |
| Model development | First we used traditional logistic regression combined with the random forest algorithm (RF) to screen the variables. The following predictors were considered to be significant risk factors for IR: BMI, TG, ALT, and FPG. these variables constitute model 1.  Next, we screened the combination of variables based on the BSR method: when the model contains 4 variables, BMI, Course, FPG, and ALT, the minimum BIC reaches -167.16911 as the optimal inflection point. Therefore, these 4 variables constitute model 2.  After comparing predictive accuracy, discrimination, and clinical utility, we finally determined that Model 1 was the best model.(Fig.4) |
| Model specification and performance | We developed a predictive nomogram containing BMI (OR 1.43, 95% CI: 1.33-1.54), TG (OR 1.22, 95% CI: 1.02-1.52), ALT (OR 1.03, 95% CI: 1.01-1.04), and FPG (OR 5.19, 95% CI: 2.98-9.04) to predict the risk of PCOS combined with IR (Fig.5.A).  We calculated the final score for each patient in the entire dataset based on nomograms, risk-stratified the total score using the cut function, and categorized patients into 3 risk groups: low-risk, medium-risk, and high-risk. Compared with the low-risk group, the intermediate-risk group was more than 9 times more likely to have an IR event (OR = 9.14, 95% CI: 5.66-15.14), whereas the outcome was more severe in the high-risk group (OR = 58.63, 95% CI: 29.09-75.46). All results were statistically significant (P< 0.001), indicating the prognostic value of risk stratification in the overall data set. |

**Appendix Table 1.** Missing observations and imputation methods for model features

| Characteristics | Missing observations, no. (%) | Imputation method |
| --- | --- | --- |
| Age | 0 | NA |
| Heredity | 23(4.0) | Logistic Regression |
| Course | 0 | NA |
| BMI | 16(2.8) | Predictive Mean Matching |
| UML | 5(0.8) | Predictive Mean Matching |
| FPG | 0 | NA |
| AMH | 19(3.3) | Predictive Mean Matching |
| T | 0 | NA |
| SHBG | 34(5.9) | Predictive Mean Matching |
| TC | 28(4.9) | Predictive Mean Matching |
| TG | 28(4.9) | Predictive Mean Matching |
| HDL | 28(4.9) | Predictive Mean Matching |
| LDL | 28(4.9) | Predictive Mean Matching |
| AST | 6(1.0) | Predictive Mean Matching |
| ALT | 6(1.0) | Predictive Mean Matching |
| SUA | 26(6.3) | Predictive Mean Matching |
| VD | 89(15.5) | # |

*NA, not applicable.

#This indicator was not included in the final variables due to missing values greater than 15 percent.
